# Individualised benefit–harm balance of aspirin as primary prevention measure – a good proof-of-concept, but could have been better…

**DOI:** 10.1186/s12916-016-0648-9

**Published:** 2016-07-06

**Authors:** Mangesh A. Thorat

**Affiliations:** Centre for Cancer Prevention, Wolfson Institute of Preventive Medicine, Queen Mary University of London, Charterhouse Square, London, EC1M 6BQ UK; Breast Services, Division of Surgery and Interventional Science, Whittington Hospital, Magdala Avenue, London, N19 5NF UK

**Keywords:** Aspirin, Prevention, Cancer, Cardiovascular disease, Benefit–harm balance, Bleeding, Gastrointestinal, Modelling, Personalised medicine

## Abstract

Guidelines from different organisations regarding the use of aspirin for primary prevention vary despite being based on similar evidence. Translating these in practice presents a further major challenge. The benefit–harm balance tool developed by Puhan et al. (*BMC Med* 13:250, 2015) for aspirin can overcome some of these difficulties and is therefore an important step towards personalised medicine. Although a good proof-of-concept, this tool has some important limitations that presently preclude its use in practice or for further research. One of the major benefits of aspirin that has become apparent in the last decade or so is its effect in preventing cancer and cancer-related deaths. However, this benefit is clear and consistent in randomised as well as observational evidence only for specific cancers. Additionally, it has long lag-time and carry-over periods. These nuances of aspirin’s effects demand a specific and a more sophisticated model such as a time-varying model. Further refinement of this tool with respect to these aspects is merited to make it ready for evaluation in qualitative and quantitative studies with the goal of clinical utility.

Please see related article: http://bmcmedicine.biomedcentral.com/articles/10.1186/s12916-015-0493-2

## Background

Public health or patient management guideline recommendations are based on broad risk groups and very often take only a few dimensions into account, i.e. one or two major benefits and one or two major harms. It is therefore no surprise that recommendations of different organisations vary despite these being based on similar evidence as they may consider different dimensions or use different risk group categorisation. Guidelines for the use of aspirin in primary prevention are an excellent example of such divergent recommendations [1–5]. Further, even if there was one universal set of recommendations, translating these recommendations in practice would still present a major challenge not only because the categorisation in broad risk groups is often too crude to apply to an individual but also because an individual’s clinical profile often has several more dimensions to consider in addition to those which formed the basis of the applicable recommendations. Furthermore, individual preferences and perceptions often fall outside the scope of guidelines, and yet these are a very important component of the ultimate informed decision which must occur at an individual level. This underscores the need to develop tools or methods to aid the in-depth analysis of the benefit–harm balance at an individual’s level in a way that also incorporates personal preferences. The benefit–harm balance tool for aspirin developed by Puhan et al. [6] is therefore one such valuable step in our quest for personalised medicine.

## Aspirin’s effects are site-specific and time-varying

This benefit–harm balance tool [6] is a good proof-of-concept but has some important limitations that need to be highlighted. An important caveat about any model is that it can only be as good as its underlying assumptions – this is where the nuances of aspirin’s effects matter. A large body of evidence [7–12] now exists which shows that it takes approximately 3 and 5 years for aspirin’s effect on cancer incidence and cancer-related deaths, respectively, to become apparent. There is also a 5-year carry-over benefit on cancer incidence and at least 10 years for cancer deaths following cessation of aspirin use. Puhan et al. [6] used the Gail/National Cancer Institute approach [13] for their modelling, however, considering such long periods of lag as well as carry-over benefit, time-varying modelling would have been more appropriate.

Puhan et al. [6] also modelled aspirin’s effects on 12 cancers based on data from randomised controlled trials (RCTs) reported by Algra and Rothwell [14]. Data from RCTs are most robust for pre-specified primary endpoints. Barring the Women’s Health Study [10], none of the RCTs that have so far been reported had cancer as their primary endpoint and Women’s Health Study data were not included in the analyses by Algra and Rothwell [14]. Despite these analyses, as well as others by Rothwell et al. [11, 12, 15–17], being robust, they should be considered in the context of data from observational studies [18]. After a thorough review of the evidence [8], we concluded that a clear and large benefit exists for colorectal, oesophageal and stomach cancer, and the benefit for lung, breast and prostate cancer is smaller and less clear. Many aspirin experts agree with a beneficial effect on only three gastrointestinal (GI) tract cancers due to the biological and pharmacological plausibility of such an effect [19], as well as due to some uncertainty regarding aspirin’s effects on lung, breast and prostate cancer. Therefore, we provided sensitivity analyses with aspirin’s beneficial effect being limited to three GI cancers as well as colorectal cancer alone [8]. Recent analyses by the U.S. Preventive Services Task Force also take into account only the beneficial effect on colorectal cancer [20]. Therefore, assuming aspirin’s effect on cancers other than colorectal, oesophageal, stomach, lung, breast and prostate cancers is not correct, even when simulations and repetitions consider the statistical uncertainty of effect on other cancers.

The flexibility to adjust weights as per individual preference is a major strength of Puhan et al’s study [6]. However, they assigned a default weight of 1.0 for GI bleeding, which is very misleading. Many end-users will often go by the default weights and it is therefore important to get this right. Their default weight was based on 3-year survival of 45.5 % in one study [21]. GI bleeding is often an accompanying sign or sequelae of a major illness and long-term survival is largely driven by the original disease. Unlike myocardial infraction, stroke or cancer, GI bleeding rarely results in a long-term morbidity on its own. Therefore, using the same approach of 5-year survival to derive weight for GI bleeding is not appropriate and use of 30-day mortality to determine default weight would have been more appropriate. We have extensively reviewed 30-day mortality in GI bleeding (any bleeding), and despite the increasing mortality risk with age, it does not exceed 10 % even in older individuals [22]. Furthermore, these mortality rates continue to fall with improving standards of care [22]. Finally, although aspirin without doubt increases the risk of GI bleeding, it has not been shown to significantly increase the risk of fatal GI bleeding [23, 24]. In short, a default weight of 0.1 would have been more appropriate than that of 1.0.

The authors discuss some of the limitations discussed above, although these should not have existed in the first place, even for a proof-of-concept study. A more thorough approach in reviewing the current evidence as well as an in-depth understanding of the nuances of aspirin’s benefits and harms would have eliminated most of these limitations, making this conceptually excellent tool ready for the next steps of qualitative and quantitative research studies to assess the clinical utility of such an approach.

## Future directions and conclusions

Puhan et al. [6] have demonstrated a good proof-of-concept and the computational feasibility of such benefit–harm balance tool. Once refined, as discussed above, this tool can then be subjected to further research, including research in supplementary preference-eliciting tools and presentation formats as discussed by the authors. The clinical utility of such a refined tool will also need to be evaluated in clinical trials. While further research in such tools continues, two important areas also merit simultaneous attention. This tool is based on the incidence of cardiovascular, cancer and bleeding events. Many individuals and their clinicians are keen to know an intervention’s impact on saving lives. Therefore, a similar tool based on mortality due to these diseases should also be developed. The tool also needs to be regularly updated as new reliable evidence becomes available. If the clinical utility of such a tool is demonstrated and the tool gets updated regularly, it will greatly enhance our ability to deliver personalised medicine not only by estimating the benefit–harm balance at an individual level, based on a range of factors in that individual’s clinical profile, but also by taking that person’s individual preferences into account. This would create a vital link between public health and individualised medicine, thus enabling personalised public health.

## Abbreviations

GI, Gastrointestinal; MI, Myocardial Infraction; RCTs, Randomised Controlled Trials; WHS, Women’s Health Study
